# Comparing the monoisocentric and dual isocentric techniques in chest wall radiotherapy of mastectomy patients

**DOI:** 10.1120/jacmp.v16i1.5069

**Published:** 2015-01-08

**Authors:** Amin Banaei, Bijan Hashemi, Mohsen Bakhshandeh

**Affiliations:** ^1^ Department of Medical Physics Faculty of Medical Sciences, Tarbiat Modares University Tehran Iran; ^2^ Department of Radiology Faculty of Allied Medical Sciences, Shahid Beheshti University of Medical Sciences Tehran Iran

**Keywords:** breast cancer, radiotherapy planning, monoisocentric technique, dual isocentric technique, radiation measurements

## Abstract

The monoisocentric (MIT) and dual isocentric (DIT) techniques are compared for the mastectomy patients undergoing chest wall radiotherapy, and a new practical method is suggested for determining the dose calculation reference point to be used in the MIT. Data of 18 mastectomy patients having chest wall radiotherapy were used. To find the appropriate dose calculation reference point for the MIT, the target tissue was divided into nine regions with 17 points as the appropriate candidates. After finding the best reference point for the MIT, dose calculations were made for each patient based on the MIT and DIT to determine the dose distributions of the target volume and organs at risk. The lateral component of the dose calculation reference point was found to be located at one‐third of the distance between the geometrical center and the lateral border of the chest wall in the lateral direction toward the outer border. The longitudinal component of this point was found to be located at the geometrical center of the chest wall with a depth located around 2–3 cm under the patients’ skin. There was no significant difference between the two radiotherapy planning techniques (MIT and DIT) regarding the dose distributions in the organs at risk and the 95% of the prescribed dose coverage of the target tissue. However, a significant difference for the 105% of the prescribed dose coverage, maximum dose delivered to the target tissue, and the level 2 lymph nodes dose was found, with the DIT showing higher values. Because of the good matching and no superposition observed between the treatment fields in the MIT, it was expected and confirmed that the hot and cold regions (with higher and lower doses than the prescribed dose) with the MIT are significantly fewer than that of the DIT. Therefore, to perform a better conformal radiotherapy for the patients having mastectomy, it could be recommended to use the MIT instead of the DIT and other conventional techniques.

PACS numbers: 87.53.Bn, 87.53.Kn, 87.55.D‐, 87.55.ne

## I. INTRODUCTION

Radiotherapy of the cancer patients after mastectomy and lumpectomy is performed to increase the chance of conserving and protecting the remaining breast in lumpectomy cases and also prevent the local recurrence of the cancer in different areas of the chest wall.^1^, ^2^

Two methods are commonly used in three‐dimensional conformal radiotherapy (3D CRT), including the MIT and the DIT. Because of the overlaps of the treatment fields encountered in the DIT, the regions with higher or lower doses than the prescribed doses appeared at the junctions of treatment fields. On the other hand, in the MIT, the dose distribution cannot be normalized to the isocenter point, since this point is located under the jaw edge of the linear accelerator collimator. Therefore, one of the most important problems required to be solved in the MIT is to find an appropriate dose calculation reference point for it.

In an investigation^(3)^ in which an MIT was introduced for the patients undergoing the breast radiotherapy (without mastectomy), a dose calculation reference point was proposed without giving any explanation/reason for choosing its location. They compared their proposed MIT with a traditional DIT. The patients were placed in supine position on the tilt board. The board was inclined until the patients’ chest wall was visually parallel to the table top. The physician then marked the position of the matchline, the medial, and lateral dorsal beam edge entrance points on the patient's skin. On the contour graph, a line was drawn connecting the medial and lateral dorsal beam edge entry points. Finally, a perpendicular line was drawn from this dorsal line, bisecting the treatment volume. The proposed dose calculation reference point was positioned on the perpendicular line and was further dictated by the field width, chosen to allow 1.5–2 cm flash beyond the anterior extent of the breast (chest wall).

In another study,^(4)^ the dose calculation reference point was placed in the midplane of the breast where the isocenter of the tangential fields in the DIT was located. Then, the true isocenter was placed in the same coordinates as the dose calculation point, except its Y component (at both of the up and down directions). It was claimed that it would be more beneficial for the dose distribution and treatment planning if the true isocenter and dose calculation point have the same X and Z coordinates.

In another investigation^(5)^ with no introduction and discussion regarding the dose calculation reference point, several treatment planning techniques were studied for irradiating the cervical spinal cord and breast in which a three‐field technique, similar to the MIT, was used for the breast and supraclavicular irradiation with half beams. The authors attained several equations for finding the gantry, collimator, and treatment couch angles for tangential fields where the isocenters were assumed to be located at specific points. Some equations were introduced for finding an isocenter when the field apertures were assumed to be constant.

In another study,^(6)^ the dose distributions in the breast and organs at risk (lung and heart) were assessed with the MIT compared with a traditional matching technique. It was reported that the dose values in the fields’ junction areas are more than the prescribed doses and, in some regions in a tumor, the dose is under the 95% of the prescribed dose with the traditional technique. On the other hand, with the MIT, the hot and cold areas claimed to disappear and the doses to the organs at risk and normal tissues decreased.

Furthermore, for the patients who didn't have a mastectomy, a general formula for exact geometric matching in the radiotherapy of the breast and supraclavicular fossa is presented.^(7)^ The proposed method does not require additional shielding to eliminate divergence other than the four independent jaws.

Other investigators^8^, ^9^, ^10^ compared various dose calculation algorithms of treatment planning systems (TPSs) with each other and used Monte Carlo calculations as the reference. In these studies, 6 and 18 MV photon beams were compared for different tissues having quite different densities like the bone, soft tissues, and lungs. The best agreement reported with the Monte Carlo results was for the collapsed cone convolution (CCC) TPS algorithm.

However, to our knowledge, there is no investigation presenting any technique or equation for finding the appropriate dose calculation reference point with the MIT for the patients having a mastectomy despite their anatomical analogies. In addition, there is no investigation in which the two common conventional treatment techniques (MIT and DIT) used for the chest wall radiotherapy of mastectomy patients would have been compared with each other. Hence, the purpose of this study was to fill the above gaps encountered in clinical situations by applying the above radiotherapy techniques on relevant patients and compare the outcomes based on the dose distributions in the target and critical organs.

## II. MATERIALS AND METHODS

Data of 18 mastectomy patients were used in this study, having a mean age of 52 years and ranging from 34 to 69 years old.

### A. Computer treatment planning

The computer treatment planning system used in this study was the Isogray (Version 4.1.64) produced by the Dosisoft Company in France in 2013.

The algorithm used for dosimetry calculations was the CCC, since previous studies^8^, ^9^, ^10^ have confirmed it as an appropriate algorithm to be used in breast cancer radiotherapy in which the effect of inhomogeneities is also considered.

Patients were placed in supine position on the linac bed, while their ipsilateral arms were elevated by using a breast board. Images with 1 mm resolution taken from the patients with a CT simulator were acquired and exported to the computer TPS database in the DICOM format. The target tissue of the patients defined by the radiotherapy oncologist was located at the mastectomy region. The lymph nodes were irradiated with a supraclavicular field with the same prescribed dose as that of the target tissue. The prescribed dose was 50 Gy delivered to the target tissue of the patients in 25 fractions.

First, the DIT plan was designed (in three dimensions) with the wedges by using two tangential fields each one delivering 25 Gy (50% of the prescribed dose) to the mastectomy location on the chest wall, and one supraclavicular field delivering 50 Gy to the regional lymph nodes located at the local levels of 1, 2, and 3. Then, one isocenter was assumed for both of the tangential fields and another isocenter for the supraclavicular field, each considered as their dose normalization points. The isocenter of the tangential fields was usually located at the same distance from the superior and inferior border of the target tissue and at a depth which typically ranged from 2 cm to 3 cm from the surface of the chest wall; it was increased up to 4.5 cm for some of the patients with thicker chest walls. The isocenter of the supraclavicular field was usually located at the center of the field and at a depth of 2 cm to 3 cm from the patient's skin.

**Figure 1:**
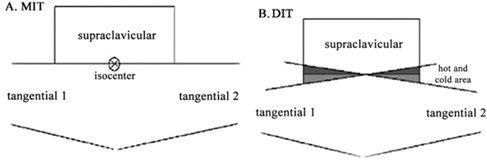
The MIT (a) and DIT (b) treatment fields showing the occurrence of hot and cold areas in the DIT

**Figure 2:**
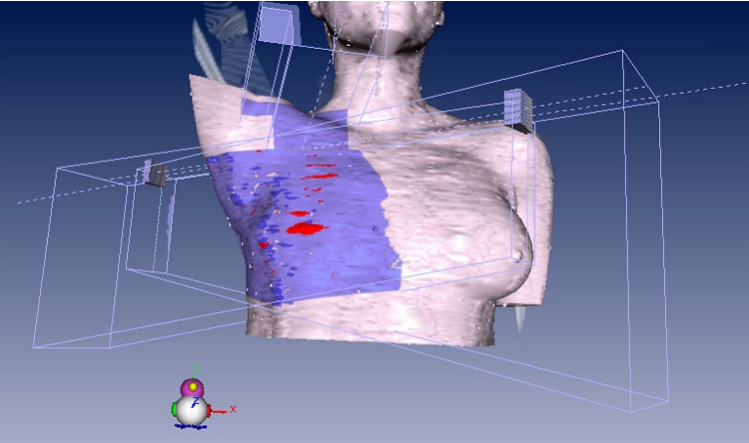
An example of the treatment planning configuration used for the MIT.

**Figure 3:**
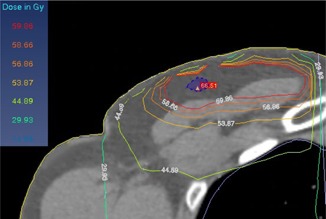
An example of the dose distributions obtained by the TPS for the DIT in the region where the tangential and supraclavicular fields overlap (in the transverse plane).

**Figure 4:**
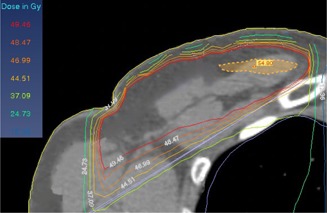
An example of the dose distributions obtained by the TPS for the MIT in the region where the tangential and supraclavicular fields collapse with each other (in the transverse plane).

For the MIT, the isocenter point was placed at the end of the superior edge (with the same distance from the lateral and medial borders) of the chest wall. The tangential and supraclavicular fields were then set by this isocenter. When the tangential fields were set, the superior half of the beam was closed while, for the supraclavicular field setting, the inferior half of the field was closed by the collimator jaw. With this condition no divergence or overlapping occurs (Fig. 1(a)). An example of the treatment planning configuration used for the MIT is shown in Fig. 2. Two examples of the patient dose distributions derived from the TPS with the DIT and MIT where the tangential and supraclavicular fields interface with each other by either overlapping or collapsing at the transverse plane are shown in Figs. 3 and 4, respectively.

### B. Finding the dose calculation reference point for the MIT

**Figure 5:**
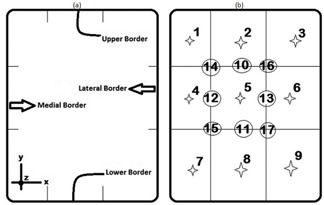
A schematic figure showing the locations of the borders defined for a left breast on the chest wall at the coronal plane (a) and the regions showing the calculation reference point candidates (b). The edges of the three irradiation beams have been the same as shown in Fig. 2.

The superior, inferior, lateral, and medial borders of the mastectomy location were defined by the radiotherapy oncologist. These borders (located at the X‐Y plane) with a thickness of tissues located above the lung (at the Z direction) specified the chest wall. Then, a coronal plane at the middle of the maximum thickness of the chest wall was chosen and divided into nine regions, as illustrated in Fig. 5. After planning the treatment fields, the dose calculation reference point was assumed to be located at every one of these regions and their boundaries with the central region. The outer boundaries of the regions were not studied since the doses would have been higher than the limited values where the dose calculation reference point is very far away from other regions in the target. The reference depth position was found to be located around 2–3 cm beneath the chest wall depending on the thickness of the patients’ chest walls.

For defining the location of these regions, the longitudinal component (Y) of the superior and inferior borders of the chest wall was attained. The distance between the borders was divided by 2 to determine the Y component of the central point. Likewise, the (X) lateral component of the central point of the chest wall in the X‐Y midplane was determined. Then, the final rectangle area was divided into nine regions (Fig. 5).

The dose calculation reference point must not be placed in the bones, under a shield, or in the lungs for the supraclavicular field. In such cases it will be better to place the dose calculation reference point at the center of an open field and a depth ranged from 2 cm to 3 cm.

### C. Dosimetric parameters

We introduced a parameter, named K, representing the distance between the geometrical center of the midplane of the chest wall and its lateral border.

**Figure 6:**
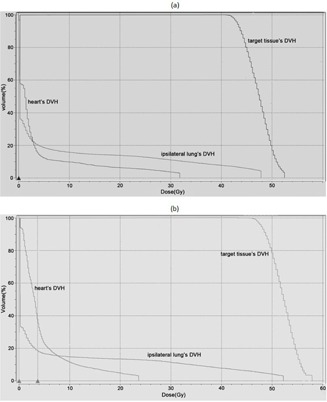
An example of the DVH of the target tissue and organs at risk calculated for one of the patients with the MIT (a) and DIT (b).

Some other parameters were required to be assessed for evaluating the dose distributions. These parameters derived from relevant dose volume histograms (DVH) included the $V_{95\%}$ (the percentage of the target tissue volume irradiated to ≥95% of the prescribed dose), the $V_{105\%}$ (the percentage of the target tissue volume irradiated with ≤105% of the prescribed dose), the $V_{20\%}$ and $V_{30\%}$ (the percentage of the ipsilateral lung volume receiving 20 Gy and 30 Gy doses, respectively), and finally the $V_{10\%}$ and $V_{40\%}$ (the percentage of the heart volume receiving 10 Gy and 40 Gy doses, respectively). Figure 6 show an example of the DVHs plots of the target tissue and organs at risk for one of the patients with the MIT (a) and DIT (b). In addition, the mean doses delivered to the lymph nodes were assessed.

### D. Statistical analysis

Relevant statistical tests were performed by using the SPSS software (ver.16). The nonparametric Kolmogrov‐Smirnov test was initially performed to determine the normality of data distributions. The paired sample *t*‐test was then performed to examine significant difference between the MIT and the DIT results for every dosimetric parameter of interest.

## III. RESULTS

The point providing the closest dose to the 100% of the prescribed dose to the target tissue (with 95% confidence interval), as well as the least dose to the organs at risk (the ipsilateral lung and heart), was regarded as the best candidate for the dose calculation reference point. The points meeting these criteria were the point number 13 and 12 for the patients with the right and left breast mastectomy, respectively (Fig. 5).

For the patients having either right or left breast mastectomy, the Y component of the dose calculation reference point was the same as that of the geometrical center point. The X component of the dose calculation reference point for all the patients was located at one‐third of the length of the K toward the lateral border of the chest wall. The depth of the reference point was determined at 2–3 cm under the patient skin.

The results of the Kolomogrov‐Smirnov test showed that all the dosimetric parameters follow the normal distribution with a 95% confidence interval, enabling us to use required parametric statistical tests.

Table 1 presents the results of statistical tests used to examine the significant differences between the two techniques for one parameter with a 95% confidence interval. Differences are meaningful when the “significant” (Sig.) value is smaller than 0.05.

Table 2 presents the means and ranges of the dosimetric parameters resulted from the implementation of both of the MIT and the DIT for the treatment planning of the patients. It must be noted that the dosimetric parameters reported in the table for the heart as a critical organ are calculated just for the patients having the left breast mastectomy (10 women).

As can be noted from the data presented in Tables 1 and 2, all the dosimetric parameters show no significant differences between the MIT and the DIT, except the three parameters of the “maximum dose in the fields junctions”, “$V_{105\%}$ for the target tissue” and “the mean dose of the level 2 lymph nodes”, indicating quite significant lower values for the MIT.

**Table 1:** The statistical test results showing significant differences of dosimetric parameters between the MIT and DIT performed on the mastectomy patients undergoing chest wall radiotherapy

*Dosimetric Parameter*	*Sig. Value* ^a^ *(2‐tailed)*	*Difference (mean)*	*Standard Deviation*	*Standard Error (mean)*
The lung mean dose (Gy)	0.34	0.64	2.81	0.66
$V_{20\%}$ ^b^	0.69	−0.76	5.48	2.91
$V_{30\%}$ ^b^	0.25	−1.77	5.68	3.39
The heart mean dose^c^ (Gy)	0.28	0.63	7.36	0.55
$V_{10\%}$ ^c^	0.21	9.41	5.66	1.44
$V_{40\%}$ ^c^	0.86	−0.22	4.01	1.26
$V_{95\%}$ ^d^	0.99	0.01	6.32	4.89
$V_{105\%}$ ^d^	0.00	−10.63	8.93	2.11
The maximum dose in the fields’ junctions (Gy)	0.00	−9.86	4.88	1.15
The level 1 lymph nodes mean dose (Gy)	0.200	3.45	5.73	2.34
The level 2 lymph nodes mean dose (Gy)	0.006	−3.90	2.11	0.86
The level 3 lymph nodes mean dose (Gy)	0.015	−2.66	1.79	0.73

a A value derived from performing the *t*‐test that is regarded to be statistically significant if it will be smaller than 0.05.

b For the ipsilateral lung.

c For the patients having left breast mastectomy.

d For the target tissue.

**Table 2:** The means and ranges of the calculated dosimetric parameters resulting from the MIT and DIT on the mastectomy patients undergoing chest wall radiotherapy

	*MIT*		*DIT*	
*Dosimetric Parameter*	*Mean*	*Range*	*Mean*	*Range*
The lung mean dose (Gy)	13.0	9.2–19.3	13.7	9–19.4
$V_{20\%}$ ^a^	26.6	19.9–38.1	27.2	20.4–37
$V_{30\%}$ ^a^	22.9	14.3–34.1	24.6	15–34.8
The heart mean dose^b^ (Gy)	3.5	0.6 – 7.0	3.2	0.6–6.8
$V_{10\%}$ ^b^	11.5	5.1–18.9	9.6	0.0–17.9
$V_{40\%}$ ^b^	5.1	3.2–14.2	5.3	0.0–13.5
$V_{95\%}$ ^c^	81.3	70.0–92.3	81.3	65–93
$V_{105\%}$ ^c^	8.8	0.0–22.1	19.5	0.0–49.8
The maximum dose in the fields’ junctions (Gy)	52.7	48.9–60.1	62.5	56.8–77.2
The level 1 lymph nodes mean dose (Gy)	47.4	42.3–52.4	43.9	34.6–50.4
The level 2 lymph nodes mean dose (Gy)	47.1	42.4–51.3	51.0	48.9–53.4
The level 3 lymph nodes mean dose (Gy)	46.5	42.4–50.4	49.2	45.6–51.8

a For the ipsilateral lung.

b For the patients having left breast mastectomy.

c For the target tissue.

## IV. DISCUSSION

The treatment fields in the MIT are very similar to the DIT when the mastectomy patients are treated at supine position. Significant differences are noted in the regions of the field junctions, collimator, and treatment couch angles. Therefore, it was expected that there would be no noticeable difference between these techniques for the $V_{95\%}$ parameter as these regions do not have a large volume compared with the entire volume of the target tissue (chest wall). Results of a study published in 2012^(6)^ reported similar results for this parameter between the MIT and traditional techniques.

As a result of using the asymmetric fields in the junction regions of the tangential and supraclavicular treatment fields in the MIT, there is a good matching and no divergence between the fields. But, in the DIT, because of using the full fields, there is a divergence in the region of the fields’ junctions. The fields cannot match very well in these regions and there would be an overlap of the treatment fields. Hence, it will be obvious to observe the regions with higher dose than the limited prescribed dose. Results also showed noticeable difference for the 105% dose coverage or even higher level of doses for the DIT compared with the MIT. Previous investigations have reported similar findings.^4^, ^5^, ^6^, ^7^, ^11^, ^12^, ^13^

Because of the reasons outlined above, it was expected and confirmed that the maximum dose in the fields’ junction and overlap regions for the DIT is significantly higher than the MIT. Previous investigations have also reported similar findings in this regard.^3^, ^6^, ^11^, ^13^

Despite the lack of appropriate matching between the treatment fields in the DIT, it will be hard to rotate the collimator or treatment couch to a desired angle in the MIT, because it will cause the divergence and overlap of the fields in the regions of the fields’ junctions. In patients whose chest walls are curved, a portion of the lung may be placed under the treatment field which is needed to be shielded by blocks; therefore, the dose distributions (in critical organs) will be similar for both of the MIT and DIT for some dosimetric parameters.

The level 1 and 3 lymph nodes are usually located in supraclavicular region. Therefore, as expected, the mean doses received by these nodes from our MIT and DIT didn't differ significantly. But, since the level 2 lymph nodes were located in the region of the fields’ junction, the mean dose delivered to these nodes by the MIT and DIT were significantly different with the MIT, giving a lower dose well within the dose limit (45–50 Gy) recommended for them.

## V. CONCLUSIONS

The dose distribution in the lung, heart, and the mean doses of the level 1 and 3 lymph nodes resulted from the MIT and the DIT did not indicate any significant difference. In addition, the dose distribution of the 95% of the prescribed dose coverage and the dose distributions in critical organs with the MIT were identical with that of the DIT. However, regarding the 105% dose coverage, the mean dose received by the level 2 lymph nodes and the maximum dose in the fields’ junctions were noticeably lower and clinically closer to the prescribed doses with the MIT. Therefore, to achieve a better chest wall conformal radiotherapy for the patients having mastectomy, it could be recommended to use the MIT as developed in this study, instead of the DIT and conventional radiotherapy techniques.

## ACKNOWLEDGMENTS

This research was carried out by the first author under the supervision of the second author and with the help and advice of the third author as the advisor of a project at Tarbiat Modares University. The patients’ CT images and their radiotherapy planning procedures were carried out at the Radiotherapy and Oncology Department of Shohaday‐e‐Tajrish Hospital, Tehran, Iran. Therefore, the authors express their sincere appreciation to the above institutes for their financial help and technical assistance.
